# Antiviral treatment in schizophrenia: a randomized pilot PET study on the effects of valaciclovir on neuroinflammation

**DOI:** 10.1017/S0033291723000430

**Published:** 2023-11

**Authors:** Iris Jonker, Janine Doorduin, Henderikus Knegtering, Erna van't Hag, Rudi A. Dierckx, Erik F. J. de Vries, Robert A. Schoevers, Hans C. Klein

**Affiliations:** 1 Department of Psychiatry, University of Groningen, University Medical Center Groningen, Groningen, The Netherlands; 2 Department of Nuclear Medicine and Molecular Imaging, University of Groningen, University Medical Center Groningen, Groningen, the Netherlands; 3 Lentis Mental Health Institution, Groningen, The Netherlands

**Keywords:** Cognition, herpes, PK11195, virus

## Abstract

**Background:**

Patients with schizophrenia experience cognitive impairment, which could be related to neuroinflammation in the hippocampus. The cause for such hippocampal inflammation is still unknown, but it has been suggested that herpes virus infection is involved. This study therefore aimed to determine whether add-on treatment of schizophrenic patients with the anti- viral drug valaciclovir would reduce hippocampal neuroinflammation and consequently improve cognitive symptoms.

**Methods:**

We performed a double-blind monocenter study in 24 male and female patients with schizophrenia, experiencing active psychotic symptoms. Patients were orally treated with the anti-viral drug valaciclovir for seven consecutive days (8 g/day). Neuroinflammation was measured with Positron Emission Tomography using the translocator protein ligand [^11^C]-PK11195, pre-treatment and at seven days post-treatment, as were psychotic symptoms and cognition.

**Results:**

Valaciclovir treatment resulted in reduced TSPO binding (39%) in the hippocampus, as well as in the brainstem, frontal lobe, temporal lobe, parahippocampal gyrus, amygdala, parietal lobe, occipital lobe, insula and cingulate gyri, nucleus accumbens and thalamus (31–40%) when using binding potential (BPND) as an outcome. With total distribution volume (VT) as outcome we found essentially the same results, but associations only approached statistical significance (*p* = 0.050 for hippocampus). Placebo treatment did not affect neuroinflammation. No effects of valaciclovir on psychotic symptoms or cognitive functioning were found.

**Conclusion:**

We found a decreased TSPO binding following antiviral treatment, which could suggest a viral underpinning of neuroinflammation in psychotic patients. Whether this reduced neuroinflammation by treatment with valaciclovir has clinical implications and is specific for schizophrenia warrants further research.

## Introduction

Schizophrenia is a disabling disorder that often has a chronic intermittent course (van Os & Kapur, 2009). Despite a considerable amount of research, the exact etiology of schizophrenia remains unknown. Current treatment strategies can alleviate symptoms, but cannot cure the disease. It is important to find treatments that impact causative factors.

One factor that is suggested to be relevant in schizophrenia is neuroinflammation (Najjar & Pearlman, 2015), characterized by the activation of microglia in brain parenchyma (Anthony & Pitossi, 2013). In response to threats, such as brain injury or infection, microglia become activated, initiating a neuroinflammatory process to eliminate the threat and promote tissue repair (Beumer et al., 2012) With use of Positron Emission Tomography (PET) these activated microglia can be studied. Activated microglia cells show an increase in the expression of the 18-kDA translocator protein (TSPO). The tracer isoquinoline (R)-N-11C-methyl-N-(1-methylpropyl)-1-(2-chlorophenyl) isoquinoline-3-carboxamide (11C-(R)-PK11195) is a peripheral TSPO ligand that can be used for the imaging of activated microglia cells with PET. An elevation of TSPO expression suggests activated microglia and is therefore suggestive for neuroinflammation We previously demonstrated elevation of TSPO expression in the hippocampus of schizophrenia patients during psychosis using PET (Doorduin et al., 2009). The hippocampus is part of the limbic system and important for cognitive functioning, in particular working memory (Yun, Krystal, & Mathalon, 2010) and executive functioning (Archer, Kostrzewa, Beninger, & Palomo, 2008). A large meta-analysis showed premorbid and prodromal deficits in cognitive functioning, mainly IQ, that moderately declined as the disease progressed into first episode psychosis. (Mesholam-Gately, Giuliano, Goff, Faraone, & Seidman, 2009). Cognitive impairment therefore denotes a trait marker for schizophrenia and is often associated with poor social outcome (Tandon, Nasrallah, & Keshavan, 2009).

While findings on neuroinflammation in schizophrenia are inconsistent (Marques et al., 2019; Plavén-Sigray et al., 2018) and the cause of neuroinflammation remains unknown, infectious agents are thought to play a role in the development of schizophrenia (Karlsson & Dalman, 2020). Human herpes viruses can establish persistent infections in the central nervous system and cause psychiatric symptoms (Quinn, Dalziel, & Nash, 2000), and are among the most studied infectious agents in schizophrenia. Higher blood levels of antibodies against herpes simplex virus type-1 (HSV-1) were found to be associated with cognitive impairment in patients with schizophrenia (Prasad, Watson, Dickerson, Yolken, & Nimgaonkar, 2012) and in a general population of adolescents (Jonker et al., 2014). After primary infection, HSV-1 can establish latency in the trigeminal ganglion. Reactivation of HSV-1 generally causes a cold sore, but in rare cases HSV-1 transmigrates into the brain where it can cause herpes encephalitis. Interestingly, HSV-1 has a specific affinity for the temporal cortex, including the hippocampus (Barnett, Jacobsen, Evans, Cassell, & Perlman, 1994). DNA of HSV-1 may also be present in the hippocampus of subjects without herpes encephalitis (Baringer & Pisani, 1994), not causing severe encephalitis in these cases.

The association between herpes viruses and schizophrenia has led to the initiation of anti-viral treatment studies. Studies with medication that specifically disrupt DNA replication of herpes viruses did not reduce overall symptoms and cognition in schizophrenia (Bhatia et al., 2018; Breier et al., 2019; Dickerson et al., 2009). However, to our knowledge the highest dose that has been previously administered was 2 grams of valaciclovir a day, which is probably insufficient for brain penetration of sufficient quantities (Blum, Liao, & de Miranda, 1982; Bodilsen et al., 2019; Bodilsen, Nielsen, & Whitley, 2019; Smith et al., 2010).

Based on the possible role in schizophrenia, we hypothesize that a herpes virus infection in the hippocampus contributes to the development of neuroinflammation, and consequently cognitive decline, in patients with schizophrenia. The aim of this study is to determine whether the antiviral drug valaciclovir can reduce hippocampal neuroinflammation, thereby moderating psychosis and improving cognitive performance.

## Methods and materials

### Design

The study was a randomized, double blind, placebo controlled, mono-center study in which patients fulfilling the DSM-IV criteria for a schizophrenia spectrum disorder and experiencing active psychotic symptoms were treated with valaciclovir for seven days. Participants underwent two (*R*)*-*N-[^11^C]-methyl-N-(1-methylpropyl)-1-(2-chlorophenyl)isoquinoline-3-carboxamide) ([^11^C]PK11195 PET) scans for the assessment of neuroinflammation (pre-treatment and seven days post-treatment) and an MRI scan for anatomical reference. One to three days before the pre-treatment PET scan, cognitive tasks and Positive and Negative Syndrome Scale (PANSS) (Kay, Fiszbein, & Opler, 1987) were performed, which were repeated seven days post-treatment.

Participants were randomly assigned to treatment with either valaciclovir or placebo for seven days, starting after the pre-treatment PET scan. Participants were admitted to the psychiatric department of the University Medical Center in Groningen during treatment, either for 24/7 or only during daytime. Their medication was taken under supervision of a psychiatric nurse and side effects were monitored. There was a valaciclovir free period of seven days after treatment, which ensured absence of direct (confounding) pharmacological effects, such as temporarily increase in neuroinflammation.

**Fig. 1.**
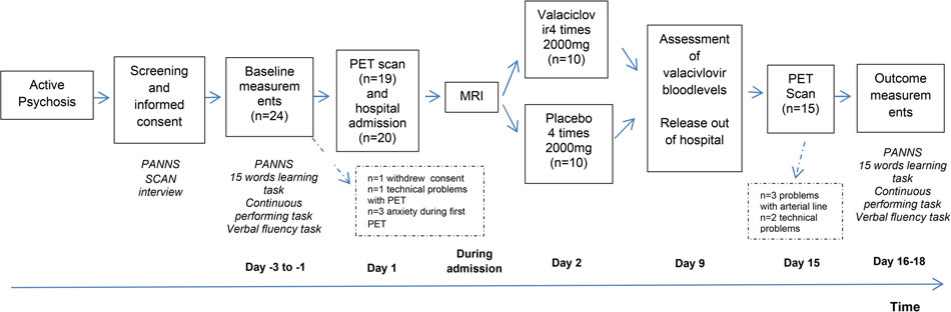
Overview of the study design. *Intervention lasted for 7 days, so day 2–9 of the study protocol.

A graphical overview of the study design is shown in Fig. 1. All study procedures were performed by three trained researchers of the UMCG.

This study was approved by the Medical Ethics Committee (institutional review board) of the University Medical Center Groningen (METc2009/105) and was registered before start of inclusion in the clinicaltrials.gov database with identifier NCT01364792 on 2 June 2011. All participants provided written informed consent and Good Clinical Practice guidelines were followed.

### Participants

Participants (*n* = 24) were recruited from either outpatient clinics or clinical settings of mental healthcare providers in the north of the Netherlands. Presence of psychosis was confirmed by a PANSS interview (Kay et al., 1987), and was defined by a score higher than 14 on the positive symptom scale, with at least 1 item scoring a 5 or 2 items scoring a 4. A Schedules for Clinical Assessment in Neuropsychiatry (SCAN) interview was used to confirm diagnosis in line with DSM IV criteria (World Health Organization, 1999). Standard laboratory tests were performed to assess kidney and liver function, in women a urine pregnancy test was performed. The exclusion criteria were: use of benzodiazepines, since they bind the same receptor as [^11^C]PK11195; use of somatic medication that may influence the immune system; use of anticoagulants; use of any investigational drug; current substance abuse; disturbed kidney function; disturbed liver function; current or recent (<4 weeks) infectious or inflammatory disease; current systemic disease; major metabolic disease; somatic, organic or neurological disorder; positive urine pregnancy test, claustrophobia; and presence of materials in the body that can be magnetized by the MRI scanner.

### Intervention

Acyclovir is an antiviral drug that is specifically phosphorylated by herpes virus thymidine kinase and incorporated into the DNA, leading to cessation of DNA synthesis of the infected cell. In case of herpes encephalitis caused by HSV-1, intravenous therapy with a dose of approximately 2 g of acyclovir per day, depending on body weight, is applied for ten days (VanLandingham, Marsteller, Ross, & Hayden, 1988). To minimize the burden of the intervention for the patients in our study, and to reach the same biological availability as in intravenous therapy, we chose to give a high dose of 8 g per day, for seven days, of the oral formulation valaciclovir, the pro-drug of acyclovir, which releases acyclovir after hydrolysis of the valine ester by esterases. This dosage is considered safe and sufficient for the long-term prevention of CMV infection after organ transplantation (Bodilsen et al., 2019; Fiddian, Sabinm, & Griffiths, 2002).

The participants were treated for 7 consecutive days, in the dosage of 4 times 2 g per day. Encapsulation and randomization of the study medication was outsourced to the pharmacy department of PRA Health Sciences (Groningen, the Netherlands), and quality control, blinding and de-blinding were done by the Department of Clinical Pharmacy and Pharmacology of the UMCG. All participants, researchers and health care practitioners were blind for the treatment until after the study was closed and all measurements were done.

### Outcome measures

The primary outcome measure of the study was the [^11^C]PK11195 binding potential (BP_ND_) in the hippocampus. Secondary outcome measures were the [^11^C]PK11195 BP_ND_ in other brain regions than the hippocampus, the scores on the positive symptom scale of the PANSS and cognitive tasks. Since it has been discussed that the total distribution volume (*V*_T_) is a more reliable outcome of [^11^C]PK11195 than the BP_ND_ (Plavén-Sigray et al., 2018; Varnäs, Varrone, & Farde, 2013), we additionally report the *V*_T_.

The sample size needed was estimated using the [^11^C]PK11195 binding potential from a previous study (Doorduin et al., 2009). In that study we found a significantly higher binding potential in the hippocampus of psychotic patients (2.07 ± 0.42, *n* = 7), when compared to healthy volunteers (1.37 ± 0.30, *n* = 8) (*p* = 0.004). The sample size was estimated assuming that the placebo-treated participants in the present study have a binding potential comparable to that of the patients in the previous study and that valaciclovir would cause a reduction in the binding potential of 50% (1.72 ± 0.42). Using an alpha of 0.05 and a power of 0.80, it was estimated that 12 participants per treatment group are sufficient to find a significant difference.

### Assessment of [^11^C]PK11195 binding

#### PET procedure

A catheter was inserted in the radial artery for arterial blood sampling after testing for collateral blood flow and injection of 1% lidocaïne. A venous catheter was placed in the antebrachial vein of the other arm, for injection of [^11^C]PK11195. PET scans were performed on an ECAT EXACT HR+ camera (Siemens Healthcare, Germany) and a Biograph mCT (Siemens Healthcare, Germany), as the HR+ was replaced during the study. The pre- and post-treatment scans of each individual participant were performed on the same camera. A head-restraining adhesive band minimalized head movement. A neuroshield was used to minimize the interference of radiation from the subject's body. After positioning in the PET camera, [^11^C]PK11195, produced as described earlier (Doorduin et al., 2009), was injected intravenously at a speed of 0.5 ml/s (total volume 8.3 ml, injected dose 378 ± 34 MBq, molar activity >12 000 GBq/mmol). Simultaneously a 60-min emission scan in 3-dimensional mode was obtained. After [^11^C]PK11195 injection, arterial blood radioactivity was continuously monitored with an automated blood sampling system (Veenstra Instruments, Joure, The Netherlands). Five extra blood samples were collected manually at 10, 20, 30, 45, and 60 min after [^11^C]PK11195 injection to determine the amount of radioactivity in the blood and plasma, to calibrate the sampling system. The arterial blood samples that were collected at 20, 45 and 60 min after tracer injection were also used for analysis of the percentage of intact tracer in plasma and to generate a metabolite-corrected plasma curve, according to the procedure described previously (De Picker, Morrens, Chance, & Boche, 2017; Doorduin et al., 2009).

T1-weighted MRI scans of the brain were made using a 3 T Intera MRI scanner (Philips, The Netherlands), and were examined for abnormalities by an experienced neuroradiologist.

#### Data analysis

The list-mode data from the PET scans were reconstructed by filtered back projection into 21 successive frames (6 × 10 s, 2 × 30 s, 3 × 1 min, 2 × 2 min, 2 × 3 min, 3 × 5 min, and 3 × 10 min). Attenuation correction was performed by the separate ellipse algorithm (ECAT EXACT HR + ) or by a low-dose CT (Biograph mCT). PMOD (version 3.8; PMOD Technologies LLC) was used for image processing and pharmacokinetic modeling. The summed PET images of each participant (frames 1–21) were aligned to the MRI scan of the same participant. Hereafter, a 6-tissue probability map normalization of the individual MRI scan into the Montreal Neurological Institute (MNI) space was performed (Ashburner & Friston, 2005). Based on the normalization, predefined volume of interest (VOIs) as based on the Hammers atlas (Hammers et al., 2003) were transformed into individual PET space. For each VOI a time-activity curve was created for pharmacokinetic modeling.

Two-tissue compartment modeling was used to calculate binding potential (BP_ND_) and the total distribution volume (*V*_T_), using the metabolite-corrected plasma curve as the input function. The BP_ND_ was defined as *k*_3_/*k*_4_ and the *V*_T_ was defined as *K*_1_/*k*_2_ × (1 + *k*_3_/*k*_4_), and were calculated for each VOI individually. To determine TSPO binding, the BP_ND_ is in principle preferred, as it is more directly related to the TSPO concentration in tissue then *V*_T_ values, as these can be affected by plasma protein binding and non-specific binding in the brain. However, for TSPO it can be challenging to reliably estimate *k*_3_ and *k*_4_, hence both the BP_ND_ and *V*_T_ are reported.

Correction for the individual delay and an individually fitted whole blood volume was applied. It was assumed that the distribution volume of the non-displaceable compartment (*K*_1_/*k*_2_) and the dissociation rate (*k*_4_) from the specific binding site was equal for all VOIs. A coupled fitting was performed that calculated a common *K*_1_/*k*_2_ and *k*_4_ for all VOIs, but a separate *K*_1_ and *k*_3_ for individual VOIs.

### Assessment of clinical functioning

#### Symptom severity

The PANSS was used to confirm inclusion criteria and to assess symptom severity before and after treatment.

#### Cognitive functioning

Cognitive functioning was assessed by a trained research physician or a research psychologist, using three cognitive tests, the 15 Word Learning Test (WLT), the Continuous Performance Test (CPT) and the Verbal Fluency Test (VFT). Impairments on these tests were found in schizophrenia patients (Nuechterlein et al., 2008).

First, the 15 WLT was used to assess memory function (Brand & Jolles, 1985), using the total number of words in the three immediate recall trials as the outcome measure (Van der Elst, van Boxtel, van Breukelen, & Jolles, 2005). Second, the unmasked CPT was used to test sustained attention (Hsieh et al., 2005). Several studies showed that A–X errors (target letter missed) and B–X errors (target letter gave a response after the wrong cue-letter) had high internal consistency among patients with schizophrenia and are therefore reliable outcome measures (Henderson et al., 2012), together with the reaction times (Birkett et al., 2007). For the VFT participants had to sum up as many words as possible from a semantic or phonetic category in 60 s. Animals and the letter N were used for the first assessment, and occupations and the letter A for the second assessment. The number of words was used as the outcome measure, representing verbal memory (Tyburski, Sokolowski, Chec, Pelka-Wysiecka, & Samochowiec, 2015).

### Statistical analysis

Statistical analysis was performed in IBM SPSS Statistics 23. Data are reported as mean ± standard deviation. Differences in general information of the participants were assessed using ANOVA (age and duration of illness), Pearson's χ^2^ test (diagnosis) and Fisher's exact test (sex, ethnicity and high education level), depending on the assumptions needed for the tests. Generalized Estimating Equations was used for the PET data, and scores on the PANSS and cognitive tests, to account for repeated measures (i.e. pre- and post-treatment) and missing data. Only subjects that underwent both scans were included in analysis on PET results. The independent correlation matrix was selected for the analysis and the Wald test used to report *p* values. Significance for our tests was reached when the *p* value was <0.05.

## Results

### Participants

Of the 24 participants, 3 withdrew because of anxiety during the pre-treatment PET scan, and 1 withdrew consent before the pre-treatment PET scan. Twenty participants (placebo, *n* = 10; valaciclovir, *n* = 10) underwent the intervention and the clinical measurements, of which 13 successfully completed the pre- and post-treatment scan (placebo, *n* = 6; valaciclovir, *n* = 7). Five participants only underwent the pre-treatment PET scan (placebo, *n* = 3; valaciclovir, *n* = 2), because of failure to place the arterial cathether (*n* = 3) and technical issues (*n* = 2) during the second scan. Two patients were excluded because of problems with both the pre- and post-treatment scan, either due to corrupt blood sampling files (2 scans), a technical issue or the post-treatment scan being delayed for technical reasons. Only the 13 subjects that underwent both scans were included in analysis on PET results. For the analysis of the general characteristics, the PANSS and cognitive tests, all 20 patients that underwent the intervention were included.

There were no statistically significant differences in the general characteristics between the valaciclovir and the placebo group (Table 1).

**Table 1.** Descriptive characteristics of study population (*n* = 20)

	Placebo (*n* = 10)	Valaciclovir (*n* = 9)	*p* value
Age (years)	35.4 ± 10.1	34.2 ± 4.7	0.739
Male (*n*)	8	7	1.000
Dutch ethnicity (*n*)	9	9	1.000
High education level^#^ (*n*)	5	6	1.000
Duration of illness (years)	9.4 ± 8.1	9.9 ± 6.6	0.882
Diagnosis (*n*) (Code 295.xx)			
Schizophrenia	8	8	
Schizoaffective	2	1	
Schizophreniform	0	0	
Medication use			
Modern antipsychotics	9	8	
Classic antipsychotics	1	1	
Antidepressant addition	4	3	
Other addition	3	2	

^#^Senior general secondary education, pre-university education, higher professional education or university education.

### Side effects

No serious adverse effects (SAE) were reported during this study. Table 2 summarizes side effects that participants recognized from a checklist. Seven participants reported temporary side effects of the arterial cathether, including bruises, pain, tingling in the fingers and distress in relation to the procedure.

**Table 2.** Side effects related to valaciclovir treatment

	Placebo	Valaciclovir
	(*n* = 10)	(*n* = 10)
Mild gastro-intestinal (nausea, diarrhea)	1	4
Dyspnea	1	0
Dizziness	1	2
Tiredness	4	3
Headache mild	4	3
Headache severe	0	1
Confusion	3	0
Depression	3	0
Mild rash	0	1
Increase of weight	1	0

### [^11^C]PK11195 binding in hippocampus

**Fig. 2.**
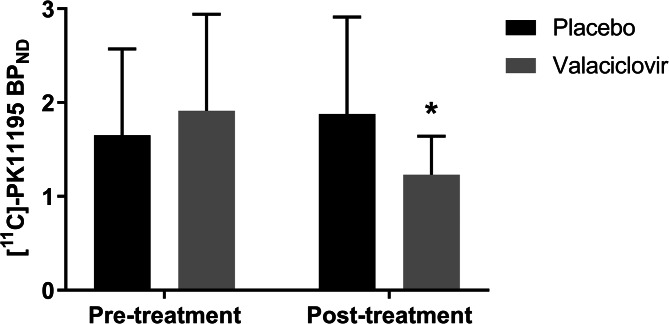
Pre- and post-treatment [11C]-PK11195 binding potential in the hippocampus. *p = 0.013 for post-treatment when compared to pre-treatment for the valaciclovir-treated groups.

The pre-treatment [^11^C]PK11195 BP_ND_ in the hippocampus did not differ between the placebo- (1.61 ± 0.93) and valaciclovir-treated (1.91 ± 1.03) participants (*p* = 0.52) (Fig. 2). No pre-treatment difference in [^11^C]PK11195 BP_ND_ was found for the two PET scanner types (HR + , 1.97 ± 1.08; mCT 1.34 ± 0.58; *p* = 0.23).

The post-treatment hippocampal [^11^C]PK11195 BP_ND_ of the valaciclovir-treated participants (1.16 ± 0.39), was not different from the BP_ND_ of the placebo-treated participants (1.88 ± 1.03) (*p* = 0.10). Within-group comparison revealed a statistically significant lower (39%) hippocampal [^11^C]PK11195 BP_ND_ for the post-treatment scan (1.16 ± 0.39), when compared to the pre-treatment scan (1.91 ± 1.03), in the valaciclovir-treated participants (*p* = 0.01). For the placebo-treated participants, the hippocampal [^11^C]PK11195 BP_ND_ was 17% higher in the post-treatment scan (1.88 ± 1.03) when compared to the pre-treatment scan (1.61 ± 0.93), but this difference was not statistically significant (*p* = 0.50).

Analyses with *V*_T_ as outcome gave essentially the same results (see Table 4), but the finding of lower hippocampal [^11^C]PK11195 binding in the valaciclovir group did not reach significance (*p* = 0.050).

### [11C]-PK11195 PET in other brain regions

No differences between the placebo- and valaciclovir-treated groups were found for the pre-treatment [^11^C]PK11195 BP_ND_ (*p* = 0.64 to *p* = 0.98) (Table 3) and no differences were found between scanner types (*p* = 0.09 to *p* = 0.43). No differences in the post-treatment [^11^C]PK11195 BP_ND_ were found between the placebo- and valaciclovir-treated groups (*p* = 0.06 to *p* = 0.25).

**Table 3.** Pre- and post-treatment [^11^C]-PK11195 binding potential in included brain regions

	Pre-treatment		Post-treatment	
	Placebo (*n* = 9)	Valaciclovir (*n* = 9)	Placebo (*n* = 6)	Valaciclovir (*n* = 7)
Brainstem	1.79 ± 1.00	2.24 ± 1.03	2.02 ± 0.86	1.47 ± 0.35*
Frontal Lobe	1.43 ± 0.87	1.58 ± 0.80	1.67 ± 0.92	1.00 ± 0.35*
Temporal lobe	1.41 ± 0.82	1.66 ± 0.86	1.65 ± 0.80	1.00 ± 0.34*
Parahippocampal gyrus	1.43 ± 0.78	1.73 ± 0.80	1.63 ± 0.70	1.13 ± 0.34*
Amygdala	1.49 ± 0.88	1.87 ± 0.99	1.93 ± 1.05	1.11 ± 0.29*
Parietal lobe	1.50 ± 0.91	1.61 ± 0.88	1.75 ± 1.11	0.97 ± 0.34*
Occipital lobe	1.55 ± 0.84	1.81 ± 0.97	1.89 ± 1.05	1.08 ± 0.32*
Insula en cingulate gyri	1.42 ± 0.76	1.68 ± 0.78	1.79 ± 0.93	1.04 ± 0.35*
Nucleus accumbens	1.39 ± 0.69	1.62 ± 0.57	1.53 ± 0.60	1.12 ± 0.53*
Thalamus	1.53 ± 0.55	2.10 ± 0.90	1.73 ± 0.50	1.37 ± 0.64*

* *p* < 0.05 for post-treatment when compared to pre-treatment for the valaciclovir-treated groups.

Within-group analysis revealed a statistically significant lower post-treatment [^11^C]PK11195 BP_ND_ for the valaciclovir-treated group, when compared to the pre-treatment BP_ND_, for the brainstem (34%; *p* = 0.02), frontal lobe (37%; *p* = 0.03), temporal lobe (40%; *p* = 0.02), parahippocampal gyrus (35%; *p* = 0.02), amygdala (40%; *p* = 0.02), parietal lobe (40%; *p* = 0.02), occipital lobe (40%; *p* = 0.02), insula and cingulate gyri (38%; *p* = 0.02), nucleus accumbens (31%; *p* = 0.03) and thalamus (35%; *p* = 0.02). In contrast, for the placebo-treated group, the [^11^C]PK11195 BP_ND_ was on average 14% higher post-treatment, which was not statistically significant [brainstem (11%; *p* = 0.50), frontal lobe (14%; *p* = 0.53), temporal lobe (14%; *p* = 0.48), parahippocampal gyrus (9%; *p* = 0.45), amygdala (19%; *p* = 0.28), parietal lobe (15%; *p* = 0.58), occipital lobe (18%; *p* = 0.42), insula and cingulate gyri (17%; *p* = 0.30), nucleus accumbens (10%; *p* = 0.71) and thalamus (10%; *p* = 0.41)].

Similar to the BP_ND_, no differences between the placebo- and valaciclovir-treated groups were found for the pre-treatment [^11^C]PK11195 V_T_ (*p* = 0.64 to *p* = 0.98) (Table 4) and no differences were found between scanner types (*p* = 0.33 to *p* = 0.89). However, the post-treatment [^11^C]PK11195 *V*_T_ was found to be statistically significantly lower in the valaciclovir-treated group when compared to the placebo-treated group, for the temporal lobe (39%; *p* = 0.044), parahippocampal gyrus (34%; *p* = 0.047), amygdala (41%; *p* = 0.048) and insula and cingulate gyri (41%; *p* = 0.042).

**Table 4.** Pre- and post-treatment [11C]- PK11195 distribution volume in included brain regions

	Pre-treatment		Post-treatment	
	Placebo (*n* = 9)	Valaciclovir (*n* = 9)	Placebo (*n* = 6)	Valaciclovir (*n* = 7)
Brainstem	0.94 ± 0.31	1.00 ± 0.56	1.05 ± 0.86	0.70 ± 0.19
Frontal Lobe	0.82 ± 0.27	0.80 ± 0.44	0.94 ± 0.92	0.56 ± 0.15
Temporal lobe	0.81 ± 0.25	0.83 ± 0.47	0.93 ± 0.80	0.56 ± 0.16#
Parahippocampal gyrus	0.82 ± 0.27	0.84 ± 0.45	0.90 ± 0.70	0.60 ± 0.15#
Amygdala	0.85 ± 0.29	0.88 ± 0.48	1.01 ± 1.05	0.60 ± 0.18#
Parietal lobe	0.84 ± 0.28	0.81 ± 0.47	0.96 ± 1.11	0.55 ± 0.15
Occipital lobe	0.86 ± 0.27	0.88 ± 0.52	1.01 ± 1.05	0.59 ± 0.16
Insula en cingulate gyri	0.84 ± 0.30	0.83 ± 0.44	0.98 ± 0.93	0.57 ± 0.16#
Nucleus accumbens	0.81 ± 0.26	0.82 ± 0.44	0.90 ± 0.60	0.58 ± 0.16*
Thalamus	0.87 ± 0.27	0.96 ± 0.56	0.96 ± 0.50	0.64 ± 0.19

^#^*p* < 0.05 for valaciclovir when compared to placebo; *p < 0.05 for post-treatment when compared to pre-treatment for the valaciclovir-treated groups.

As for the BP_ND_, the within-group analysis of the valaciclovir-treated group revealed a lower post- than pre-treatment [^11^C]PK11195 *V*_T_ (29–33%; *p* = 0.059 to *p* = 0.084), although this was only statistically significant for the nucleus accumbens (29%; *p* = 0.044). For the placebo-treated group, the [^11^C]PK11195 *V*_T_ was on average 13% higher post-treatment, which was not statistically significant (*p* = 0.360 to *p* = 0.586).

### Clinical outcomes

For the PANSS, the 15 WLT and the CPT, no pre-treatment differences were found between the placebo- and valaciclovir-treated groups (*p* > 0.05). A statistically significant higher pre-treatment score on the phonetic category of the VFT was found for the valaciclovir-treated group (14.0 ± 3.6), when compared to the placebo-treated group (10.1 ± 3.6) (*p* = 0.01). No differences were found in the score on the semantic category. No differences between the placebo- and valaciclovir treated groups were found for the post-treatment PANSS and cognitive tasks.

Within-group comparisons revealed no differences in the scores on the PANSS and the CPT (*p* > 0.05). For the valaciclovir-treated group, a statistically significant lower score on the 15 WLT was found post-treatment (22.0 ± 6.7), when compared to pre-treatment (26.2 ± 5.1) (16%, *p* = 0.01). A lower score post-treatment than pre-treatment was also observed in the placebo-treated group, but this was not statistically significant (11%, *p* = 0.11). For the semantic, but not phonetic, category of the VFT, a lower score was found post-treatment compared to pre-treatment, for both the placebo- (17.1 ± 5.0 *v.* 24.1 ± 4.6; 29%, *p* < 0.001) and valaciclovir-treated (16.2 ± 6.0 *v.* 24.7 ± 7.6; 34%, *p* = 0.001) groups.

## Discussion

To our knowledge, this is the first study investigating the effect of valaciclovir treatment on neuroinflammation in the hippocampus of patients with schizophrenia. Our main finding is that valaciclovir reduced neuroinflammation in the hippocampus, which could be interpreted as support for the hypothesis that actively replicating herpes viruses are present in the hippocampus and contribute to an inflammatory response. We additionally found significantly reduced neuroinflammation in other included brain regions. Despite the reduction in neuroinflammation, psychotic symptoms and cognitive functioning did not improve in valaciclovir-treated patients. Contrary to what was expected, the score on the 15 WLT decreased over time in valaciclovir-treated patients only, and scores on verbal fluency in both valaciclovir- and placebo-treated patients.

We hypothesized the main treatment effects of valaciclovir to be in the hippocampus, because of the affinity of HSV-1 for that region and our earlier finding of neuroinflammation in the hippocampus (Doorduin et al., 2009).

Following valaciclovir treatment we found reduction in TSPO binding for all brain regions included, which could suggest a global presence of actively replicating herpes viruses, an unknown effect of valaciclovir on TSPO binding or natural variation in neuroinflammation. We found an effect in all tested brain regions, without any clinical effects, which could be an argument for natural variation. TSPO binding was increased in the post-treatment scan, when compared to the pre-treatment scan, in the placebo group. Although this was not statistically significant, it suggests that TSPO binding varies over time. The parietal and occipital cortex that showed a reduced BP_ND_ after valaciclovir treatment, have been found to be involved in focal extended herpes simplex encephalitis (Fernandes et al., 2010). Additionally, in a postmortem study focusing on brains of individuals who died from non-neurological causes, HSV genomic sequences were detected in the medulla, olfactory bulbus, pons, gyrus rectus, amygdala and hippocampus (Baringer & Pisani, 1994). The involvement of other brain regions than the hippocampus is therefore in line with findings in herpes simplex encephalitis and the presence of herpes viruses in the brain without clinical features. Earlier studies in Alzheimer's disease found that mild HSV-1 infections could be a risk factor for the development of Alzheimer's disease, and importantly suggest that a mild, asymptomatic form of herpes infection across the brain exists, affecting cognitive functioning (Marcocci et al., 2020). Schizophrenia patients have been found to have a higher risk to develop Alzheimer's disease (Cai & Huang, 2018).

A recent meta-analysis reported on the findings of 12 PET imaging studies in schizophrenia (190 schizophrenia patients and 200 healthy controls), using TSPO as a marker of neuroinflammation (Marques et al., 2019). In schizophrenia patients, a moderate increase in TSPO tracer binding in whole brain gray matter was found when compared to controls when BP_ND_ was used as an outcome measure, but not when *V*_T_ was used. Another meta-analysis focused on frontal cortex, temporal cortex, and hippocampus and found lower *V*_T_ in schizophrenia patients when compared to healthy controls (Plavén-Sigray et al., 2018). Although we did not compare the BP_ND_ to healthy controls in our treatment study, the reduction we found suggests that neuroinflammation was indeed present.

There is some discussion on which outcome is the most reliable to determine TSPO binding. The BP_ND_ is often preferred, provided that the standard error of outcome is less than 25%, since the *V*_T_ values are more vulnerable to be affected by plasma protein binding and non-specific binding in the brain. However, for TSPO it can be challenging to reliably estimate *k*_3_ and *k*_4_, which is why we reported both outcomes (Varnäs et al., 2013). In our within-group comparisons, using *V*_T_ as the outcome revealed the same trends as for BP_ND_, but they did not reach statistical significance (although *p* = 0.05 for *V*_T_ in hippocampus). In contrast, in four brain regions a statistically significant lower post-treatment *V*_T_ was found in the valaciclovir-treated group than the placebo-treated group, which was not observed for the BP_ND_ (although *p* values were around 0.06 for these regions). In our study using the BP_ND_ or *V*_T_ as the outcome measure leads to the same conclusion, namely that valaciclovir reduced neuroinflammation. The seemingly discrepancy that the BP_ND_ reveals within-group differences and the *V*_T_ between-group differences, might be resolved with increasing the group size resulting in within- and between-group differences for both the BP_ND_ and *V*_T_.

We found a lower performance for the 15 WLT in valaciclovir-treated patients and for the semantic category of the VFT for both treatment groups. This is unusual, as cognitive scores most often improve across a study due to a learning effect. Participation in this study may have been stressful and tiring, affecting concentration in our participants. Impaired concentration could also be a side effect of the high dose valaciclovir, even though we had a wash-out period of 7 days before follow-up measurement. Furthermore, VFT scores decreased in both the valaciclovir and the placebo group. In contrast, the psychiatric condition of patients did not change, as was shown by the pre- and post-treatment PANSS scores.

We used [^11^C]-PK11195 as a PET tracer for imaging TSPO expression in neuroinflammation. Despite the small sample size we had due to missing data, we did observe a treatment effect on the [^11^C]PK11195 BP_ND_. We found no improvement in the PANSS and cognitive performance, which could be because the post-treatment measurement was relatively quick (three weeks) after the first measurement, and we did not perform a follow up at a later time point. This choice was based on the known course of a herpes infection and the fast reaction of herpes viruses to treatment. However, additional factors may have a role in the perpetuation of psychosis and cognitive impairment. We did not have sufficient data on herpes antibodies to perform analysis of the impact of presence of viral antibodies on the outcome measures, which limits the interpretation of our findings. Therefore we do not know whether the effect would be more prominent in a selection of patients who actually had herpes simplex antibodies. It could also be true that a prolonged treatment period would yield better clinical results. On the other hand, it is possible that viral infection is an irreversible causal factor, especially after a longer illness duration. This could explain the lack of effect on clinical symptoms, and would mean that valaciclovir could still have effect on symptoms in patients with a first episode of psychosis. Furthermore, we do not know whether our findings are specific for schizophrenia patients, as we did not include a healthy control group. Lastly, an important issue to raise is the choice of the demanding procedure for the participants, especially the arterial blood sampling. To reliably assess the binding potential of [^11^C]-PK11195, it is necessary to perform kinetic modeling using an arterial input function. The patient burden in this study was an important reason for the difficult and protracted patient inclusion, and for the premature drop-out of some patients. Other studies have used a reference tissue approach to avoid arterial blood sampling, but this is much less robust (Schuitemaker et al., 2007).

In conclusion, we found a decrease in TSPO binding in the hippocampus of patients with schizophrenia treated with valaciclovir, which was also observed in all other tested brain regions. Although this clearly is not proven by our study, it might suggest that viral activity is responsible for neuroinflammation in patients with schizophrenia. However, other explanations for this association cannot be excluded. Cognitive performance was negatively affected by the treatment, possibly due to study-induced stress. Additional studies are needed to confirm our findings, to find out whether this treatment effect is specific for schizophrenia patients, or for schizophrenia patients that are seropositive for HSV-1 infection, and to understand whether this treatment effect can lead to clinical relevant improvement of symptoms in patients with schizophrenia.

## References

[ref1] Anthony, D. C., & Pitossi, F. J. (2013). Special issue commentary: The changing face of inflammation in the brain. Molecular and Cellular Neurosciences, 53, 1–5. doi: 10.1016/j.mcn.2012.11.005 23147112

[ref2] Archer, T., Kostrzewa, R. M., Beninger, R. J., & Palomo, T. (2008). Cognitive symptoms facilitatory for diagnoses in neuropsychiatric disorders: Executive functions and locus of control. Neurotoxicity Research, 14(2–3), 205–225. doi: 10.1007/BF03033811 19073427

[ref3] Ashburner, J., & Friston, K. J. (2005). Unified segmentation. NeuroImage, 26(3), 839–851. doi: S1053-8119(05)00110-2 15955494 10.1016/j.neuroimage.2005.02.018

[ref4] Baringer, J. R., & Pisani, P. (1994). Herpes simplex virus genomes in human nervous system tissue analyzed by polymerase chain reaction. Annals of Neurology, 36(6), 823–829. doi: 10.1002/ana.410360605 7998767

[ref5] Barnett, E. M., Jacobsen, G., Evans, G., Cassell, M., & Perlman, S. (1994). Herpes simplex encephalitis in the temporal cortex and limbic system after trigeminal nerve inoculation. The Journal of Infectious Diseases, 169(4), 782–786. doi: 10.1093/infdis/169.4.782 8133092

[ref6] Beumer, W., Gibney, S. M., Drexhage, R. C., Pont-Lezica, L., Doorduin, J., Klein, H. C., … Drexhage, H. A. (2012). The immune theory of psychiatric diseases: A key role for activated microglia and circulating monocytes. Journal of Leukocyte Biology, 92(5), 959–975. doi: 10.1189/jlb.0212100 22875882

[ref7] Bhatia, T., Wood, J., Iyengar, S., Narayanan, S. S., Beniwal, R. P., Prasad, K. M., … Nimgaonkar, V. L. (2018). Emotion discrimination in humans: Its association with HSV-1 infection and its improvement with antiviral treatment. Schizophrenia Research, 193, 161–167. doi: S0920-9964(17)30467-X 28830742 10.1016/j.schres.2017.08.001 PMC5818324

[ref8] Birkett, P., Sigmundsson, T., Sharma, T., Toulopoulou, T., Griffiths, T. D., Reveley, A., & Murray, R. (2007). Reaction time and sustained attention in schizophrenia and its genetic predisposition. Schizophrenia Research, 95(1), 76–85. doi: 10.1016/j.schres.2007.05.030 17630256

[ref9] Blum, M. R., Liao, S. H., & de Miranda, P. (1982). Overview of acyclovir pharmacokinetic disposition in adults and children. American Journal of Medicine, 73(1A), 186–192. doi: 10.1016/0002-9343(82)90088-2 7048911

[ref10] Bodilsen, J., Nielsen, H., & Whitley, R. J. (2019). Valaciclovir therapy for herpes encephalitis: Caution advised. Journal of Antimicrobial Chemotherapy, 74(6), 1467–1468. doi: 10.1093/jac/dky568 30668736

[ref11] Brand, N., & Jolles, J. (1985). Learning and retrieval rate of words presented auditorily and visually. The Journal of General Psychology, 112(2), 201–210. doi: 10.1080/00221309.1985.9711004 4056765

[ref12] Breier, A., Buchanan, R. W., D'Souza, D., Nuechterlein, K., Marder, S., Dunn, W., … Dickerson, F. B. (2019). Herpes simplex virus 1 infection and valacyclovir treatment in schizophrenia: Results from the VISTA study. Schizophrenia Research, 206, 291–299. doi: S0920-9964(18)30632-7. 30478008 10.1016/j.schres.2018.11.002

[ref13] Cai, L., & Huang, J. (2018). Schizophrenia and risk of dementia: A meta-analysis study. Neuropsychiatric Disease and Treatment, 14, 2047–2055. doi: 10.2147/NDT.S172933 30147318 PMC6095111

[ref14] De Picker, L. J., Morrens, M., Chance, S. A., & Boche, D. (2017). Microglia and brain plasticity in acute psychosis and schizophrenia illness course: A meta-review. Frontiers in Psychiatry, 8, 238. doi: 10.3389/fpsyt.2017.00238 29201010 PMC5696326

[ref15] Dickerson, F. B., Stallings, C. R., Boronow, J. J., Origoni, A. E., Sullens, A., & Yolken, R. H. (2009). Double blind trial of adjunctive valacyclovir in individuals with schizophrenia who are seropositive for cytomegalovirus. Schizophrenia Research, 107(2–3), 147–149. doi: 10.1016/j.schres.2008.10.007 19008077

[ref16] Doorduin, J., de Vries, E. F., Willemsen, A. T., de Groot, J. C., Dierckx, R. A., & Klein, H. C. (2009). Neuroinflammation in schizophrenia-related psychosis: A PET study. Journal of Nuclear Medicine: Official Publication, Society of Nuclear Medicine, 50(11), 1801–1807. doi: 10.2967/jnumed.109.066647 19837763

[ref17] Fernandes, A. F., Lange, M. C., Novak, F. T., Zavala, J. A., Zamproni, L. N., Germiniani, F. M., … Teive, H. A. (2010). Extra-temporal involvement in herpes simplex encephalitis. Journal of Clinical Neuroscience: Official Journal of the Neurosurgical Society of Australasia, 17(9), 1221–1223. doi: 10.1016/j.jocn.2010.02.007 20541415

[ref18] Fiddian, P., Sabinm, C. A., & Griffiths, P. (2002). Valacyclovir provides optimum acyclovir exposure for prevention of cytomegalovirus and related outcomes after organ transplantation. Journal of Infectious Diseases, 186(Suppl 1), S110–S115. doi: 10.1086/342965 12353195

[ref19] Hammers, A., Allom, R., Koepp, M. J., Free, S. L., Myers, R., Lemieux, L., … Duncan, J. S. (2003). Three-dimensional maximum probability atlas of the human brain, with particular reference to the temporal lobe. Human Brain Mapping, 19(4), 224–247. doi: 10.1002/hbm.10123 12874777 PMC6871794

[ref20] Henderson, D., Poppe, A. B., Barch, D. M., Carter, C. S., Gold, J. M., Ragland, J. D., … MacDonald III, A. W. (2012). Optimization of a goal maintenance task for use in clinical applications. Schizophrenia Bulletin, 38(1), 104–113. doi: 10.1093/schbul/sbr172 22199092 PMC3245586

[ref21] Hsieh, P. C., Chu, C. L., Yang, Y. K., Yang, Y. C., Yeh, T. L., Lee, I. H., & Chen, P. S. (2005). Norms of performance of sustained attention among a community sample: Continuous performance test study. Psychiatry and Clinical Neurosciences, 59(2), 170–176. doi: PCN1353 15823163 10.1111/j.1440-1819.2005.01353.x

[ref22] Jonker, I., Klein, H. C., Duivis, H. E., Yolken, R. H., Rosmalen, J. G., & Schoevers, R. A. (2014). Association between exposure to HSV1 and cognitive functioning in a general population of adolescents. the TRAILS study. PloS One, 9(7), e101549. doi: 10.1371/journal.pone.0101549 24983885 PMC4077793

[ref23] Karlsson, H., & Dalman, C. (2020). Epidemiological studies of prenatal and childhood infection and schizophrenia. Current Topics in Behavioral Neurosciences, 44, 35–47. doi: 10.1007/7854_2018_87 30852763

[ref24] Kay, S. R., Fiszbein, A., & Opler, L. A. (1987). The positive and negative syndrome scale (PANSS) for schizophrenia. Schizophrenia Bulletin, 13(2), 261–276. doi: 10.1093/schbul/13.2.261 3616518

[ref25] Marcocci, M. E., Napoletani, G., Protto, V., Kolesova, O., Piacentini, R., Li Puma, D. D., … De Chiara, G. (2020). Herpes simplex virus-1 in the brain: The dark side of a sneaky infection. Trends in Microbiology, 28(10), 808–820. doi: 10.1016/j.tim.2020.03.003 32386801

[ref26] Marques, T. R., Ashok, A. H., Pillinger, T., Veronese, M., Turkheimer, F. E., Dazzan, P., … Howes, O. D. (2019). Neuroinflammation in schizophrenia: Meta-analysis of in vivo microglial imaging studies. Psychological Medicine, 49(13), 2186–2196. doi: 10.1017/S0033291718003057. 30355368 PMC6366560

[ref27] Mesholam-Gately, R., Giuliano, A. J., Goff, K. P., Faraone, S. V., & Seidman, L. J. (2009). Neurocognition in first-episode schizophrenia: A meta-analytic review. Neuropsychology, 23, 315–336. doi: 10.1037/a0014708 19413446

[ref28] Najjar, S., & Pearlman, D. M. (2015). Neuroinflammation and white matter pathology in schizophrenia: Systematic review. Schizophrenia Research, 161(1), 102–112. doi: 10.1016/j.schres.2014.04.041 24948485

[ref29] Nuechterlein, K. H., Green, M. F., Kern, R. S., Baade, L. E., Barch, D. M., Cohen, J. D., … Marder, S. R. (2008). The MATRICS consensus cognitive battery, part 1: Test selection, reliability, and validity. The American Journal of Psychiatry, 165(2), 203–213. doi: 10.1176/appi.ajp.2007.07010042 18172019

[ref30] Plavén-Sigray, P., Matheson, G. J., Collste, K., Ashok, A. H., Coughlin, J. M., Howes, O. D., … Cervenka, S. (2018). Positron emission tomography studies of the glial cell marker translocator protein in patients with psychosis: A meta-analysis using individual participant data. Biological Psychiatry, 84(6), 433–442. doi: S0006-3223(18)31298-8 29653835 10.1016/j.biopsych.2018.02.1171 PMC7893597

[ref31] Prasad, K. M., Watson, A. M., Dickerson, F. B., Yolken, R. H., & Nimgaonkar, V. L. (2012). Exposure to herpes simplex virus type 1 and cognitive impairments in individuals with schizophrenia. Schizophrenia Bulletin, 38(6), 1137–1148. doi: 10.1093/schbul/sbs046; 22490995 PMC3494052

[ref32] Quinn, J. P., Dalziel, R. G., & Nash, A. A. (2000). Herpes virus latency in sensory ganglia--a comparison with endogenous neuronal gene expression. Progress in Neurobiology, 60(2), 167–179. doi: 10.1016/s0301-0082(99)00024-6 10639053

[ref33] Schuitemaker, A., van Berckel, B. N., Kropholler, M. A., Kloet, R. W., Jonker, C., Scheltens, P., … Boellaard, R. (2007). Evaluation of methods for generating parametric (R-[11C]PK11195 binding images. Journal of Cerebral Blood Flow and Metabolism: Official Journal of the International Society of Cerebral Blood Flow and Metabolism, 27(9), 1603–1615. doi: 9600459 17311080 10.1038/sj.jcbfm.9600459

[ref34] Smith, J. P., Weller, S., Johnson, B., Nicotera, J., Luther, J. M., & Haas, D. W. (2010). Pharmacokinetics of acyclovir and its metabolites in cerebrospinal fluid and systemic circulation after administration of high-dose valacyclovir in subjects with normal and impaired renal function. Antimicrobial Agents and Chemotherapy, 54(3), 1146–1151. doi: 10.1128/AAC.00729-09 20038622 PMC2825963

[ref35] Tandon, R., Nasrallah, H. A., & Keshavan, M. S. (2009). Schizophrenia, “just the facts” 4. Clinical features and conceptualization. Schizophrenia Research, 110(1–3), 1–23. doi: 10.1016/j.schres.2009.03.005 19328655

[ref36] Tyburski, E., Sokolowski, A., Chec, M., Pelka-Wysiecka, J., & Samochowiec, A. (2015). Neuropsychological characteristics of verbal and non-verbal fluency in schizophrenia patients. Archives of Psychiatric Nursing, 29(1), 33–38. doi: 10.1016/j.apnu.2014.09.009 25634872

[ref37] Van der Elst, W., van Boxtel, M. P., van Breukelen, G. J., & Jolles, J. (2005). Rey's verbal learning test: Normative data for 1855 healthy participants aged 24-81 years and the influence of age, sex, education, and mode of presentation. Journal of the International Neuropsychological Society : JINS, 11(3), 290–302. doi: 10.1017/S1355617705050344 15892905

[ref38] VanLandingham, K. E., Marsteller, H. B., Ross, G. W., & Hayden, F. G. (1988). Relapse of herpes simplex encephalitis after conventional acyclovir therapy. Jama, 259(7), 1051–1053. Retrieved from https://jamanetwork.com/journals/jama/fullarticle/370702. 3339802

[ref39] van Os, J., & Kapur, S. (2009). Schizophrenia. Lancet *(*London, England*)*, 374(9690), 635–645. doi: 10.1016/S0140-6736(09)60995-8 19700006

[ref40] Varnäs, K., Varrone, A., & Farde, L. (2013). Modeling of PET data in CNS drug discovery and development. Journal of Pharmacokinetics and Pharmacodynamics, 40(3), 267–279. doi: 10.1007/s10928-013-9320-6 23660778

[ref41] World Health Organization. (1999). Schedules for clinical assessment in neuropsychiatry (version 2.1) WHO – assessment, classification and epidemiology, Geneva CH (1999).

[ref42] Yun, R. J., Krystal, J. H., & Mathalon, D. H. (2010). Working memory overload: Fronto-limbic interactions and effects on subsequent working memory function. Brain Imaging and Behavior, 4(1), 96–108. doi: 10.1007/s11682-010-9089-9 20503117 PMC2854358

